# Human Induced Pluripotent Cell‐Derived Sensory Neurons for Fate Commitment of Bone Marrow‐Derived Schwann Cells: Implications for Remyelination Therapy

**DOI:** 10.5966/sctm.2015-0424

**Published:** 2016-09-14

**Authors:** Sa Cai, Lei Han, Qiang Ao, Ying‐Shing Chan, Daisy Kwok‐Yan Shum

**Affiliations:** ^1^ School of Biomedical Sciences, University of Hong Kong, Hong Kong, People's Republic of China; ^2^ Research Centre of Heart, Brain, Hormone, and Healthy Aging, Li Ka Shing Faculty of Medicine, University of Hong Kong, Hong Kong, People's Republic of China; ^3^ Department of Neurosurgery, Yuquan Hospital, Tsinghua University, Beijing, People's Republic of China; ^4^ State Key Laboratory of Brain and Cognitive Sciences, University of Hong Kong, Hong Kong, People's Republic of China

**Keywords:** Induced pluripotent stem cells, Sensory neurons, Small‐molecule inhibitors, Schwann cells, Myelination

## Abstract

Strategies that exploit induced pluripotent stem cells (iPSCs) to derive neurons have relied on cocktails of cytokines and growth factors to bias cell‐signaling events in the course of fate choice. These are often costly and inefficient, involving multiple steps. In this study, we took an alternative approach and selected 5 small‐molecule inhibitors of key signaling pathways in an 8‐day program to induce differentiation of human iPSCs into sensory neurons, reaching ≥80% yield in terms of marker proteins. Continuing culture in maintenance medium resulted in neuronal networks immunopositive for synaptic vesicle markers and vesicular glutamate transporters suggestive of excitatory neurotransmission. Subpopulations of the derived neurons were electrically excitable, showing tetrodotoxin‐sensitive action potentials in patch‐clamp experiments. Coculture of the derived neurons with rat Schwann cells under myelinating conditions resulted in upregulated levels of neuronal neuregulin 1 type III in conjunction with the phosphorylated receptors ErbB2 and ErbB3, consistent with amenability of the neuritic network to myelination. As surrogates of embryonic dorsal root ganglia neurons, the derived sensory neurons provided contact‐dependent cues to commit bone marrow‐derived Schwann cell‐like cells to the Schwann cell fate. Our rapid and efficient induction protocol promises not only controlled differentiation of human iPSCs into sensory neurons, but also utility in the translation to a protocol whereby human bone marrow‐derived Schwann cells become available for autologous transplantation and remyelination therapy. Stem Cells Translational Medicine *2017;6:369–381*

**Significance Statement**

A highly efficient chemical protocol was developed to differentiate human induced pluripotent stem cells (iPSCs) into functional sensory neurons. The derived sensory neurons expressed membrane‐bound cues that directed bone marrow‐derived Schwann cell‐like cells to fate‐committed Schwann cells with translational prospects in autologous transplantation and remyelination therapy. The iPSC‐derived sensory neurons may also prove useful in studies of sensory neuron biology, disease modeling, and drug screening.

## Introduction

Schwann cells transplanted into the injured environments of peripheral nerve 1 and spinal cord 2 switch on an axon‐supportive program that improves nerve regeneration and functional recovery 3, 4. To take advantage of this unique plasticity of Schwann cells in autologous transplantation for therapy, sufficient numbers of such cells are required, ideally without having to sacrifice a peripheral nerve for the graft. Preliminary works with cells from rodents have in part addressed this need by in vitro derivation of Schwann cell‐like cells (SCLCs) from bone marrow stromal cells (BMSCs) 5, 6, 7, 8. The SCLCs were then directed to fate commitment via contact‐mediated signaling in coculture with neurons purified from dorsal root ganglia (DRG) of rat embryos 8. Subcultures of the derived Schwann cells 9 demonstrated robust capacity for remyelination both in vitro 8 and in vivo 10. The limiting source of human DRG neurons, however, presents a barrier to translation.

Until recently, strategies to derive functionally responsive human sensory neurons in sufficient numbers from human pluripotent stem cells have relied on complex, multistep procedures 11, 12, 13, 14, 15, 16 in recapitulation of events in the early developing neuroepithelium or neural crest cells. The advent of direct reprogramming of human fibroblasts via transcription factor overexpression heralds prospects of rapid and selective procedures to yield functionally defined classes of induced neurons 17, 18, 19, 20, 21, 22, 23. The availability of human induced pluripotent stem cells (iPSCs) and the possible use of small molecules to induce cell lineage reprogramming 24 offer the option of achieving differentiation of the iPSCs into sensory neurons. In this study, we have adapted a selection of small‐molecule inhibitors (SMIs) of key signaling pathways 25, 26 for the derivation of sensory neurons from a human iPSC line in an 8‐day program, yielding sensory neurons at ≥80% efficiency and bypassing the neural progenitor stage. The derived neurons expressed key juxtacrine signals that specify the Schwann cell fate and demonstrated amenability to myelination by Schwann cells, thus fulfilling the need for a surrogate of human DRG neurons in the translation to a protocol whereby human bone marrow‐derived Schwann cells achieve fate commitment and meet safety requirements for autologous transplantation and remyelination therapy.

## Materials and Methods

### Culture of Human iPSCs

Human iPS(IMR90) clone (no. 1) cells (WiCell Research Institute), a gift from Dr. Elly S.W. Ngan (Department of Surgery, University of Hong Kong), were maintained in culture on six‐well plates coated with human embryonic stem cell (hESC)‐qualified matrix (Stemcell Technologies, Vancouver, BC, Canada, http://www.stemcell.com) in mTeSR1 (Stemcell Technologies). The medium was changed every day. Cells were passaged using 1 mg/ml dispase (Stemcell Technologies) in Dulbecco's modified Eagle's medium (DMEM)/F‐12, washed, and replated at dilutions of 1:4 to 1:8 (supplemental online Fig. 1). Cells were monitored immunocytochemically for the ESC markers, OCT4, NANOG, SSEA3, and SSEA4.

### Generation of Sensory Neurons

**Figure 1:**
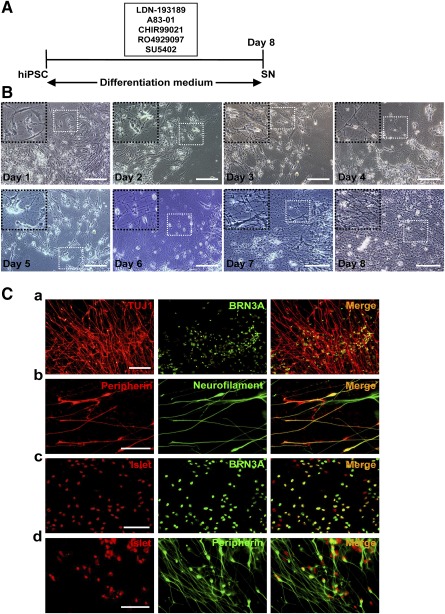
One‐step protocol for the differentiation of hiPSCs to sensory neurons. **(A):** Schematic outline of the protocol. **(B):** Morphological changes in the progression from hiPSCs to sensory neurons as viewed under phase‐contrast microscopy. Scale bars = 50 μm. **(C):** Neuronal lineage marker expression by day‐8 cells as detected by double immunofluorescence for TUJ1 and BRN3A **(Ca)**, peripherin and neurofilament **(Cb)**, Islet1 and BRN3A **(Cc)**, and Islet1 and peripherin **(Cd)**. Scale bars = 50 μm. Abbreviations: hiPSC, human induced pluripotent stem cell; SN, sensory neuron.

Human iPSC colonies at 60% confluence were dissociated using dispase and pelleted at 800 rpm for 4 minutes (room temperature). To generate sensory neurons, the cells were maintained on Matrigel‐coated plates in DMEM/F12 with defined supplements plated at a density of 1 × 10^5^ via one of three protocols. (1) Three‐step protocol: cultures were supplemented from day 1 with 10% KnockOut Serum Replacement (KSR) (Thermo Fisher Scientific Life Sciences, Waltham, MA, http://www.thermofisher.com), 1% penicillin/streptomycin (Thermo Fisher), and 0.3 μM LDN‐193189 (Cellagen Technology, San Diego, CA, http://www.cellagentech.com) to day 5 and with 2 μM A83‐01 (Cellagen Technology) to day 10; from day 5, with 6 μM CHIR99021 (BioVision, Milpitas, CA, http://www.biovision.com) to day 14; and from day 8, with 2 μM RO4929097 (Cellagen Technology) and 3 μM SU5402 (Tocris, Bristol, UK, http://www.tocris.com) to day 14. (2) Two‐step protocol: cultures were supplemented from day 1 with 10% KSR, 1% penicillin/streptomycin, 0.3 μM LDN‐193189, and 2 μM A83‐01 to day 7 and with 6 μM CHIR99021 to day 12; and from day 5, with 2 μM RO4929097 and 3 μM SU5402 to day 12. (3) One‐step protocol: cultures were supplemented from day 1 with 10% KSR, 1% penicillin/streptomycin, 0.3 μM LDN‐193189, 2 μM A83‐01, 6 μM CHIR99021, 2 μM RO4929097, and 3 μM SU5402 to day 8. Retinoic acid (0.3 μM; Sigma‐Aldrich, St Louis, MO, http://www.sigmaaldrich.com) was included in all cultures. The medium was refreshed every 2 to 3 days. The neural induction protocols are outlined in supplemental online Figures 2 and 3 and Figure 1. To test cultures for stability of phenotype, cultures were maintained in neurobasal medium supplemented with 10 ng/ml neurotrophin 3, 20 ng/ml brain‐derived neurotrophic factor, 20 ng/ml nerve growth factor (NGF), and 20 ng/ml glial cell line‐derived growth factor, with medium refreshed every other day for up to 2 weeks. Cultures were then tested for marker expression.

### Immunofluorescence

For immunostaining, cells were rinsed once with phosphate‐buffered saline (PBS), fixed with 4% paraformaldehyde, and treated with blocking buffer (1% bovine serum albumin in PBS and 0.1% Triton X‐100). Cells were then incubated overnight at 4°C with the selected primary antibodies: OCT3/4 (mouse monoclonal, 1:200; BD Biosciences, San Jose, CA, http://www.bdbiosciences.com), NANOG (mouse monoclonal, 1:200; eBioscience, San Diego, CA, http://www.ebioscience.com), SSEA3 (rat monoclonal, 1:100; BD Biosciences), SSEA4 (mouse monoclonal, 1:100; BD Biosciences), nestin (mouse monoclonal, 1:400; Abcam, Cambridge, MA, http://www.abcam.com), PAX6 (mouse monoclonal, 1:200; Santa Cruz Biotechnology, Santa Cruz, CA, http://www.scbt.com), SOX2 (rabbit monoclonal, 1:200; Abcam), p75^NTR^ (rabbit polyclonal, 1:500; Abcam), HNK1 (mouse monoclonal, 1:200; Sigma‐Aldrich), AP2 (mouse monoclonal, 1:400; Sigma‐Aldrich), TUJ1 (mouse/rabbit monoclonal, 1:500; Covance Laboratories, Dedham, MA, http://www.covance.com), neurofilament 200 (mouse monoclonal, 1:500; Covance Laboratories; rabbit monoclonal, 1:100; Cell Signaling Technology, Danvers, MA, http://www.cellsignal.com), peripherin (mouse monoclonal/rabbit polyclonal, 1:500; Abcam), BRN3A (mouse monoclonal, Santa Cruz Biotechnology 1:200; Santa Cruz Biotechnology; rabbit monoclonal, 1:200; Abcam), NeuN (mouse monoclonal, 1:500; EMD Millipore, Billerica, MA, http://www.emdmillipore.com), Islet1 (mouse monoclonal/rabbit polyclonal, 1:400; Abcam), MAP2 (rabbit polyclonal, 1:200; Abcam), vesicular glutamate transporter 1 (VGLUT1) (rabbit polyclonal, 1:100; Abcam), VGLUT2 (rabbit polyclonal, 1:400; Novus Biologicals, Littleton, CO, http://www.novusbio.com), VGLUT3 (rabbit polyclonal, 1:400; Alomone Labs, Jerusalem, Israel, http://www.alomone.com), synapsin (rabbit polyclonal, 1:500; EMD Millipore), VAMP/synaptobrevin (mouse monoclonal, 1:400; Thermo Fisher Scientific Life Sciences), myelin protein zero (P0; rabbit polyclonal, 1:200; Abcam), myelin basic protein (MBP; mouse monoclonal, 1:500; EMD Millipore), galactocerebroside (GALC; mouse monoclonal, 1:200; EMD Millipore), ErbB2 (rabbit polyclonal, 1:400; Santa Cruz Biotechnology), ErbB3 (rabbit polyclonal, 1:400; Santa Cruz Biotechnology), CD90 (mouse monoclonal, 1:200; BD Biosciences), CD73 (mouse monoclonal, 1:500; BD Biosciences), GFAP (mouse monoclonal, 1:100; Abcam), and S100 (rabbit polyclonal, 1:200; Abcam). Cells were then incubated with the appropriate secondary antibody for 2 hours at 24°C. The secondary antibodies included Alexa Fluor 488‐conjugated goat antimouse immunoglobulin G (IgG) (1:300), Alexa Fluor 594‐conjugated goat antirabbit IgG (1:300), Alexa Fluor 568‐conjugated goat antimouse IgG (1:400), and Alexa Fluor 555‐conjugated goat antirabbit IgG (1:500) (all Thermo Fisher). Nuclei were counterstained with 4,6‐diamidino‐2‐phenylindole (Abcam). Cells were viewed under an inverted fluorescence microscope (Olympus IX7).

### Flow Cytometric Analysis

Cultures were dissociated with dispase, washed in PBS, and blocked with 2% bovine serum albumin in PBS for 1 hour. The cells were fixed and then incubated at 4°C for 30 minutes with appropriate primary antibodies at 1:50 dilution. Primary antibodies were omitted in the negative controls. Double staining was performed using fluorescein isothiocyanate‐conjugated BRN3A monoclonal antibody and phycoerythrin‐conjugated peripherin monoclonal antibody (Santa Cruz Biotechnology). Flow cytometric analysis was performed with use of BD CantoII Analyzer and CellQuest software and set to record 20,000 events per sample. For cell‐cycle distribution analysis, the cells were harvested and washed with PBS and fixed with 70% ethanol for 24 hours at −20°C. After washing, fixed cells were stained for at least 30 minutes in the dark with 25 mg/ml propidium iodide solution containing 200 mg/ml RNase A.

### Western Blot Analysis

Whole cell lysates were prepared as previously described 27. Briefly, cells in culture were washed with PBS and lysed with Laemmli sample buffer containing protease and phosphatase inhibitors. Protein concentrations in sample lysates were determined by Bradford assay for equal protein loading per lane in sodium dodecyl sulfate‐polyacrylamide gel electrophoresis. Protein patterns were electroblotted onto polyvinylidene difluoride membrane (EMD Millipore). Nonspecific binding was blocked with 5% nonfat skim milk at 24°C for 2 hours, and the epitope of interest was probed overnight at 4°C. Primary antibodies used included those against neuronal neuregulin 1 (NRG1) type III (rabbit polyclonal, 1:500), ErbB2 and phospho‐ErbB2 (rabbit polyclonal, 1:500), and ErbB3 and phospho‐ErbB3 (rabbit polyclonal, 1:500) (all Santa Cruz Biotechnology). Additionally, blots were probed for β‐actin (rabbit polyclonal against β‐actin, 1:500; Abcam) as internal reference. Secondary antibodies were conjugated with horseradish peroxidase. The membranes were washed in Tris‐buffered saline with Tween 20 between incubations. Visualization was enhanced with an enhanced chemiluminescence kit (EMD Millipore). Images were captured with the ChemiImager 5500 imaging system (Alpha Innotech Co., San Leandro, CA; http://www.alphainnotech.com).

### Reverse‐Transcription Polymerase Chain Reaction

Total RNA was extracted using TRIzol reagent according to the manufacturer's instructions, followed by DNase treatment (Thermo Fisher). RNA (2 µg) was used for first‐strand complementary DNA (cDNA) synthesis. Reverse‐transcription polymerase chain reaction (RT‐PCR) was performed with 200 ng cDNA, Taq DNA polymerase, and the primers listed in supplemental online Table 1. mRNA signals were quantified by densitometric analysis using a Biometra BioDoc‐Analyzer, and the ratio of their expression to that of a housekeeping gene (GAPDH) was calculated.

### Electrophysiology

The procedures for whole‐cell patch‐clamp recordings of human iPSC‐derived sensory neurons were performed at room temperature (20–22°C) using methods described previously 28. Low‐resistance recording pipettes (2–3 MΩ) were pulled from glass capillaries. Neurons were visually identifiable by round‐to‐oval soma. Extracellular solution contained 137 mM NaCl, 5 mM KCl, 2 mM CaCl_2_, 1 mM MgCl_2_, and 10 mM glucose in 10 mM HEPES, pH 7.4. The intracellular (pipette) solution contained 115 mM K‐gluconate, 7 mM KCl, 0.05 mM EGTA, 2 mM Na_2_ATP, 2 mM MgATP, and 0.5 mM Na_2_GTP in 10 mM HEPES, pH 7.3. The osmolarity of all solutions was maintained at 290 mOsm/l for the extracellular solution and 300 mOsm/l for the intracellular solution. All chemicals were purchased from Sigma‐Aldrich. To isolate potassium currents, NaCl in the solution was replaced with *N*‐methyl‐glucamine chloride Ringer. Currents were digitized at 25 kHz and filtered at 10 kHz. Action potentials were recorded by holding the membrane potential at −60 mV and injecting increasing currents to elicit action potentials. Signals were recorded using an Axon MultiClamp 700B amplifier controlled by Clampex 10.2 software (Molecular Devices, Sunnyvale, CA, http://www.moleculardevices.com).

### Human iPSC‐Derived Sensory Neuron/Rat Schwann Cell Cocultures

Cocultures of iPSC‐derived sensory neurons and purified rat Schwann cells from neonatal rat sciatic nerves (ScienCell Research Laboratories, Carlsbad, CA, http://wwwsciencellonline.com) were prepared as described previously 8. Neurite networks of iPSC‐derived sensory neurons were seeded with purified rat Schwann cells at a density of 100,000 cells/coverslip in maintenance medium containing 10 ng/ml NGF, 5 ng/ml neurotrophin 3, 10 ng/ml brain‐derived neurotrophic factor, 10 ng/ml glial cell line‐derived growth factor, 0.08% glucose, and 10% fetal bovine serum (FBS). Medium was refreshed every 2 to 3 days. Five to 7 days later, the coculture system was supplemented with 50 μg/ml ascorbic acid (Sigma‐Aldrich), 10 ng/ml NGF, 0.08% glucose, and 10% FBS to induce myelination by Schwann cells. The medium was refreshed every 2 to 3 days for up to 2 weeks. Myelination was assessed in terms of P0‐, MBP‐, and GALC‐positive neurite segments observable under fluorescence microscopy.

### Generation of SCLCs and Coculture of SCLCs With iPSC‐Derived Sensory Neurons

Our previous study demonstrated that contact‐mediated signaling provided by embryonic DRG neurons is essential for the SCLCs to become fate‐committed Schwann cells 8. Here we asked whether the iPSC‐derived sensory neurons can serve as an alternative to the limiting source of DRG neurons. SCLCs were derived from rat BMSCs as described 8. SCLCs were seeded onto the iPSC‐derived sensory neurons at 3,000 cells/cm and maintained for 2 weeks in coculture medium: glutamine‐free α‐minimum essential medium and neurobasal medium (1:1, vol/vol) supplemented with 2.5 μM forskolin, 2.5 ng/ml platelet‐derived growth factor, 5 ng/ml basic fibroblast growth factor, 100 ng/ml β‐heregulin, 5 ng/ml NGF, 1% (vol/vol) B27, and 5% FBS. Every 2 to 3 days, 50% of the medium was replaced with fresh medium. After passaging to remove iPSC‐derived sensory neurons, the surviving cells were maintained in basal medium (DMEM/F12 with 10% FBS) for at least 1 week. Cells that stained positive for Schwann cell markers at this post‐coculture stage were termed committed Schwann cells. SCLC cultures that had not been cocultured with iPSC‐derived sensory neurons were maintained in parallel under the same conditions as controls.

### Statistical Analysis

Statistical analyses were performed using a commercially available software package (Prism 5; GraphPad, San Diego, CA, http://www.graphpad.com). Data are presented as mean ± SEM or SD as indicated. Statistical significance was assessed using Student's *t* test or nonparametric analysis of variance. All experiments were repeated at least five times.

## Results

### Derivation of Sensory Neurons From Human iPSCs

To begin with, the human iPSC colonies homogeneously showed immunoreactivities for ESC markers, OCT4, NANOG, SSEA3, and SSEA4 (supplemental online Fig. 1). In the 3‐step protocol (supplemental online Fig. 2), day‐5 cells that had been subjected to dual‐Smad inhibition with LDN‐193189 and A83‐01 showed immunopositivity for the neural progenitor cell (NPC) markers, PAX6, nestin, and SOX2. Next, via supplementation with CHIR99021 to inhibit glycogen synthase kinase‐3β and thus maintain Wnt/β‐catenin signaling, day‐8 cells showed pronounced immunopositivity for the neural crest stem cell (NCSC) markers, p75^NTR^, HNK1, and AP2. Finally, via supplementation with RO4929097 (a γ‐secretase inhibitor of Notch signaling) and SU5402 (an inhibitor of FGFR1‐specific tyrosine kinase) in the context of CHIR99021, day‐14 cells showed immunopositivity for the markers TUJ1, neurofilament, BRN3A, and Islet1, suggestive of sensory neurogenesis.

In the 2‐step protocol (supplemental online Fig. 3), day‐5 cells that had been treated in concert with LDN‐193189, A83‐01, and CHIR99021 showed pronounced immunopositivity for the NCSC markers, p75^NTR^, HNK1, and AP2. Then, under CHIR99021, RO4929097, and SU5402, day‐12 cells showed immunopositivity for markers of the sensory neuron lineage.

In the 1‐step protocol, human iPSCs that had been treated concurrently with LDN‐193189, A83‐01, CHIR99021, RO4929097, and SU5402 in an 8‐day program (Fig. 1A) showed progressive changes in morphology, from round or fusiform cells with dense and prominent nucleoli to ones with compact cell bodies and multiple processes that in time apparently formed interconnecting networks (Fig. 1B).

**Figure 2:**
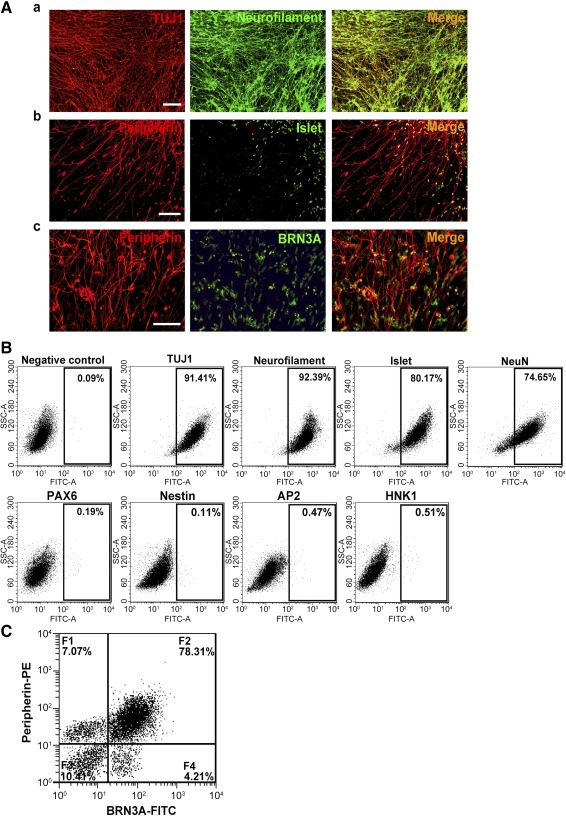
Continuing culture of human iPSC‐derived neurons in maintenance medium for 14 days. **(A):** Neuronal lineage marker expression by the iPSC‐derived neurons as detected by double immunofluorescence for TUJ1 and neurofilament **(Aa)**, peripherin and Islet1 **(Ab)**, and peripherin and BRN3A **(Ac)**. Scale bars = 50 μm. **(B):** Representative dot plots of flow cytometric data showing the percentage of iPSC‐derived cells positive for TUJ1 (91.41%), neurofilament (92.39%), Islet1 (80.17%), and NeuN (74.65%) but not for PAX6, Nestin, AP2, and HNK1 (<1%). **(C):** Representative dot plot of two‐color flow cytometric data showing subpopulations of the iPSC‐derived cells: F1, low in BRN3A but high in peripherin; F2, high in both BRN3A and peripherin; F3, low in both BRN3A and peripherin; F4, high in BRN3A but low in peripherin. Abbreviations: FITC, fluorescein isothiocyanate; iPSC, induced pluripotent stem cell; PE, phycoerythrin; SSC, side scatter.

Immunocytochemical staining showed that most of the derived cells were positive for markers of neuronal cytoskeleton, TUJ1, and neurofilament (supplemental online Fig. 4A) and neuronal nuclear antigen, NeuN (supplemental online Fig. 4B). Double immunofluorescence showed coexpression of these markers with those of the sensory neuron lineage, such as TUJ1 and BRN3A (Fig. 1Ca), peripherin and neurofilament (Fig. 1Cb), Islet and BRN3A (Fig. 1Cc), and Islet and peripherin (Fig. 1Cd). These iPSC‐derived neurons were confirmed to be immunonegative for the NPC markers, PAX6 and nestin (supplemental online Fig. 5A, 5B) as well as the neural crest cell markers, AP2, HNK1, and p75^NTR^ (supplemental online Fig. 5C–5E). Phenotypic stability of the iPSC‐derived neurons in neural maintenance medium in the absence of SMIs could be maintained for 2 weeks as indicated by immunopositivity for TUJ1 and neurofilament (Fig. 2Aa), peripherin and Islet1 (Fig. 2Ab), or BRN3A (Fig. 2Ac). Flow cytometric analysis of the iPSC‐derived neurons for TUJ1, neurofilament, Islet, and NeuN showed percentages as high as 91.41%, 92.39%, 80.17%, and 74.65%, respectively, compared with the negative control; in contrast, immunopositivity for PAX6, nestin, AP2, and HNK1 was negligible, being less than 1% (Fig. 2B). A representative dot plot of peripherin‐positive counts (*y*‐axis) against BRN3A‐positive counts (*x*‐axis) showed that 78% of the population (F2, Fig. 2C) was doubly positive for the sensory neuron markers.

**Figure 3:**
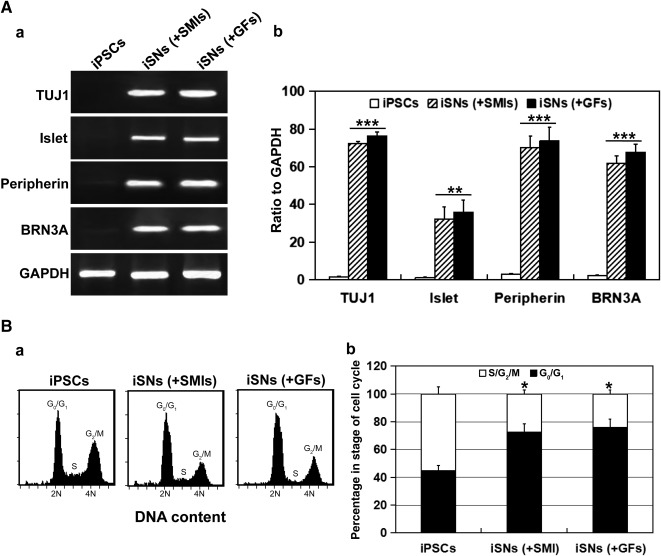
Stability of human iPSC‐derived sensory neurons in terms of marker expression and cell‐cycle profile. **(A):** Semiquantitative reverse transcription‐polymerase chain reaction analysis revealed sustained expression of mRNA for TUJ1, Islet1, peripherin, and BRN3A in iPSC‐iSNs on day 8 of SMI treatment (+SMIs) and those on day 14 of maintenance treatment with neurobasal medium and growth factors (+GFs) as shown with ethidium bromide‐stained gel patterns **(Aa)** and densitometric scans of the specific marker relative to that of GAPDH **(Ab)**. ∗∗, *p* < .01; ∗∗∗, *p* < .005, iSNs (+SMIs), iSN (+GFs) vs. iPSCs. **(B):** Cell‐cycle analysis revealed that the proportion of cells in the G_2_/M phase remained as low as those in the G_0_/G_1_ phase for iSN (+SMIs) and iSN (+GFs). ∗, *p* < .05, iSNs (+SMIs), iSN (+GFs) vs. iPSCs. Abbreviations: GAPDH, glyceraldehyde‐3‐phosphate dehydrogenase; GF, growth factor; iPSC, induced pluripotent stem cell; iSN, induced sensory neuron; SMI, small‐molecule inhibitor.

**Figure 4:**
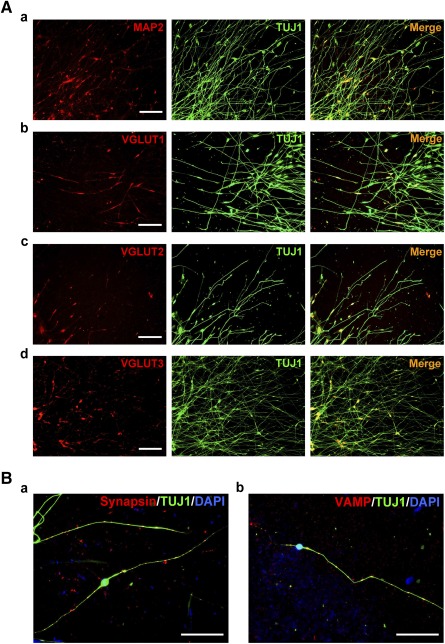
Immunodetection of synaptic vesicle‐associated proteins in human iPSC‐derived neurons. **(A):** Double immunofluorescence revealed MAP2 **(Aa)**, VGLUT1 **(Ab)**, VGLUT2 **(Ac)**, and VGLUT3 **(Ad)** in TUJ1‐positive iPSC‐derived neurons on day 14 of maintenance treatment. Scale bars = 50 μm. **(B):** Double immunofluorescence revealed synapsin **(Ba)** and VAMP **(Bb)** along neurites of iPSC‐derived neurons. Nuclei were visualized with DAPI. Scale bars = 50 μm. Abbreviations: DAPI, 4′,6‐diamidino‐2‐phenylindole; iPSC, induced pluripotent stem cell; VGLUT, vesicular glutamate transporter.

We then sought confirmation via RT‐PCR analysis for expression at the mRNA level. The set of sensory neuron markers not only was expressed among cells exposed to SMIs, but was also as highly expressed among cells in maintenance medium after withdrawal of SMIs (Fig. 3Aa). Semiquantitative analysis revealed ≥16‐fold induction of BRN3A and ≥20‐fold induction of peripherin expression in the derived neurons compared with the parental iPSCs (Fig. 3Ab). Furthermore, fluorescence‐activated cell sorting analysis found a decrease in the proportion of cells in the cell cycle (Fig. 3Ba, 3Bb), in support of the increase in proportion of cells directed to the sensory neuron lineage after treatment with the SMIs. Numerous TUJ1‐positive cells coexpressed MAP2 (Fig. 4Aa), a neuron‐specific marker protein that stabilizes microtubules in dendrites of mature neurons. Immunopositivity for VGLUT1 (Fig. 4Ab), VGLUT2 (Fig. 4Ac), and VGLUT3 (Fig. 4Ad) suggests that the neurons were in preparation for glutamatergic transmission, as supported by the finding of TUJ1‐positive cells showing immunopositivity for the synaptic vesicle proteins, synapsin (Fig. 4Ba) and VAMP (Fig. 4Bb).

### Electrophysiological Properties of iPSC‐Derived Sensory Neurons

**Figure 5:**
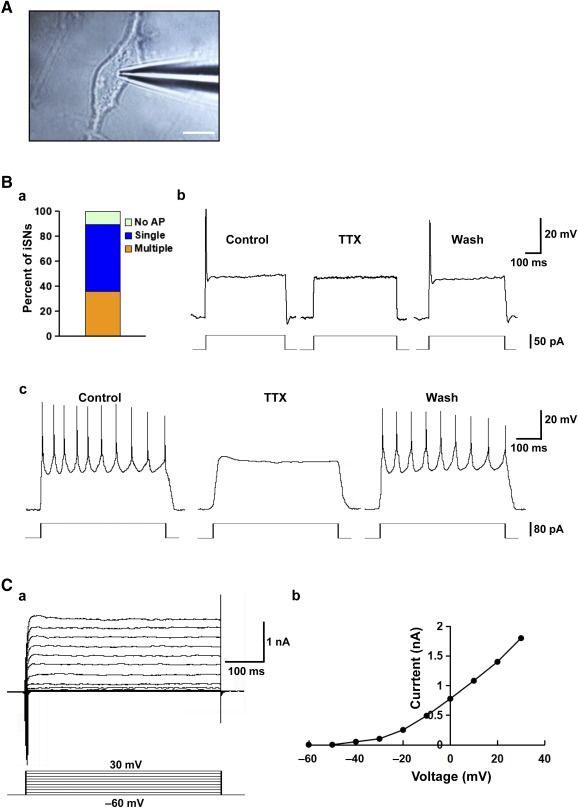
Electrophysiological properties of the human iPSC‐derived sensory neurons. **(A):** Photomicrograph showing a patch pipette attached to an iPSC‐derived neuron. Scale bar = 10 μm. **(B):** Current‐clamp recording of membrane potential in iPSC‐derived neurons: proportion of neurons showing null/single/multiple action potential firing as evoked with step‐current injection **(Ba)**; example recording of a single action potential evoked by current injection of 50 pA for 1 second **(Bb)**; and example recording of multiple action potential firing as evoked by current injection of 80 pA for 1 second **(Bc)**. Application of TTX blocked the generation of action potentials, but after washout, action potential firing could again be evoked. **(C):** Voltage‐clamp recording of specific ion currents: example recording of delayed rectifier K^+^ current evoked by depolarizing steps ranging from −60 mV to +30 mV **(Ca)**; and steady‐state current‐voltage relationship for the cell examined **(Cb)**. Abbreviations: AP, action potential; iPSC, induced pluripotent stem cell; iSN, induced sensory neuron; TTX, tetrodotoxin.

Whole‐cell patch‐clamp recordings were selectively performed on derived neurons displaying bipolar morphology (Fig. 5A). In total, we recorded 28 cells, of which 25 (89.3%) fired action potentials in response to depolarizing current pulses (Fig. 5Ba). The majority of cells (53.6%, *n* = 15) fired a single spike in response to depolarization (Fig. 5Bb). As demonstrated in Figure 5Bc, multiple spikes could be generated in a subpopulation of the derived neurons (35.7%, *n* = 10) after suprathreshold current injections. These spikes were abolished with use of 1 μM tetrodotoxin (TTX) but were recoverable after washout, suggesting that TTX‐sensitive voltage‐gated channels were in operation in the derived neurons for the generation of action potentials. In response to incremental voltage steps from −60 mV to +30 mV (10‐mV increments), 82% of the sampled neurons (*n* = 23) displayed inward fast‐inactivating sodium and a large outward current with little or no inaction, typical of a delayed, rectifying K^+^ current (Fig. 5Ca). The sustained portion of the outward current increased steadily with increasing depolarization (Fig. 5Cb). These data demonstrate that the iPSC‐derived sensory neurons possess membrane and electrophysiological properties that are characteristic of functional neurons.

### Human iPSC‐Derived Sensory Neurons Engaged in Contact‐Mediated Signaling With Rat Schwann Cells in Coculture

**Figure 6:**
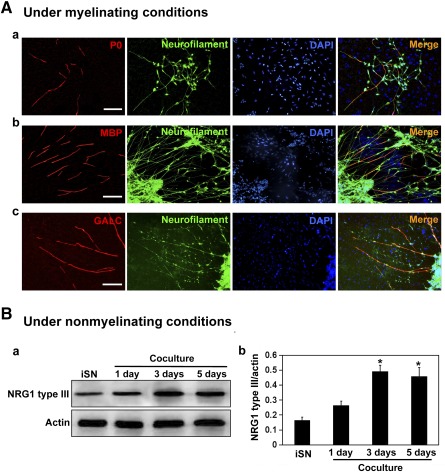
Contact‐mediated signaling between human iPSC‐derived sensory neurons and rat Schwann cells in coculture. **(A):** Images of myelin‐like segments along neuritic networks of human iPSC‐derived sensory neurons in coculture with rat Schwann cells for 14 days in medium supplemented with ascorbic acid. Double immunofluorescence for P0/neurofilament **(Aa)**, MBP/neurofilament **(Ab)**, and GALC/neurofilament **(Ac)**. Cell nuclei were visualized with DAPI. Scale bars = 50 μm. **(Ba):** Western blot analysis for NRG1 type III in cell lysates prepared from iPSC‐derived iSNs alone or iSN‐Schwann cell cocultures for the indicated days under nonmyelinating conditions. Schwann cells were given this period to populate the neurites before induction of myelination with ascorbic acid supplementation. **(Bb):** Histogram showing NRG1 type III band density as normalized against the corresponding actin band density. NRG1 type III was detectable in extracts of iSNs and at increased levels in extracts of cocultures, leveling off by day 3 of coculture **(Ba)**. ∗, *p* < .05, cocultures at days 1, 3, and 5 vs. iSNs alone. Abbreviations: DAPI, 4′,6‐diamidino‐2‐phenylindole; GALC, galactocerebroside; iPSC, induced pluripotent stem cell; iSN, induced sensory neuron; MBP, myelin basic protein; NRG1, neuregulin 1.

Myelination requires activation of NRG1 type III and ErbB signaling at the Schwann cell‐axon junction. We established the coculture model of iPSC‐derived sensory neurons with purified rat Schwann cells and determined the interaction of the two cell types after the initiation of myelination. After 3 weeks in coculture, segments that were positive for myelin proteins P0 (Fig. 6Aa), MBP (Fig. 6Ab), and GALC (Fig. 6Ac) formed along axon bundles of the derived sensory neurons. The increased level of NRG1 type III in extracts of cocultures was confirmed by Western blot analysis (Fig. 6Ba, 6Bb). ErbB2‐ and ErbB3‐positive cells (supplemental online Fig. 6Aa, 6Ab) observable along the neuritic networks of derived sensory neurons are likely Schwann cells. The observation of phosphorylated proteins *p*‐ErbB2 and *p*‐ErbB3 in Western blots (supplemental online Fig. 6Ba, 6Bb) further supports that NRG1 type III interaction with ErbB2‐ErbB3 heterodimer activated receptor cross‐phosphorylation and downstream signaling in the coculture.

### Commitment of SCLCs to Schwann Cells After Coculture With Human iPSC‐Derived Sensory Neurons

**Figure 7:**
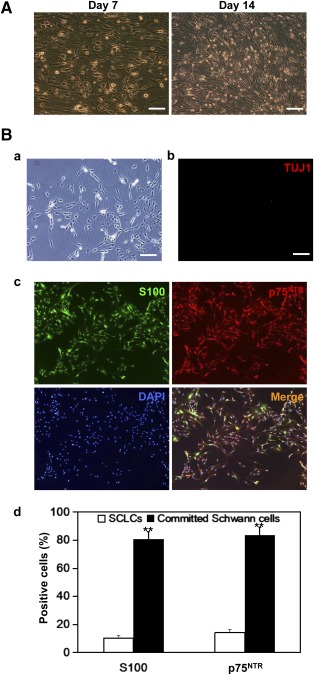
Commitment of SCLCs to Schwann cell fate after coculture with iPSC‐derived sensory neurons. **(A):** Phase‐contrast images showing SCLCs on days 7 and 14 of coculture with iPSC‐derived sensory neurons. Scale bar = 100 μm. **(B):** Fate‐committed Schwann cells subcultured in basal medium without supplementation of glia‐inducing factors as viewed under phase‐contrast microscopy **(Ba)** and fluorescence microscopy **(Bb, Bc)**. No sign of TUJ1‐positive neurons was detectable **(Bb)**, but the fate‐committed Schwann cells were positive for both S100 and p75^NTR^ **(Bc)**. Histogram showing percentages of DAPI‐stained cells showing immunopositivity for S100 and p75^NTR^ in cultures of SCLCs (open bar) versus those of fate‐committed Schwann cells (filled bar) **(Bd)**. Scale bar = 100 μm. ∗∗, *p* < .01, committed Schwann cells vs. SCLCs. Abbreviations: DAPI, 4′,6‐diamidino‐2‐phenylindole; iPSC, induced pluripotent stem cell; SCLC, Schwann cell‐like cell.

SCLCs were derived from rat BMSCs as described 8 (supplemental online Fig. 7). Seven days after coculture, SCLCs adopted typical Schwann cell morphology and organization (Fig. 7A). One week later, these cells increased in number, and a lot of cells formed a network (Fig. 7A). After passaging and withdrawal of both gliogenic cocktail and iPSC‐derived sensory neurons, the Schwann cell culture was negative for TUJ1 (Fig. 7Bb), confirming the absence of neurons. The culture of committed Schwann cells demonstrated persistence of spindle‐shaped morphology (Fig. 7Ba). Immunopositivity for Schwann cell markers S100 and p75^NTR^ (Fig. 7Bc) was observable in 80.7% ± 5.3% and 83.2% ± 6.1%, respectively, of the committed Schwann cells. In contrast, the ratios of positively stained cells in parallel SCLC cultures were as low as 10.1% ± 1.8% and 14.3% ± 2.1%, respectively (Fig. 7Bd). Taken together, these results show that the derived sensory neurons conferred the contact‐dependent cues necessary for switching SCLCs to the Schwann cell fate.

## Discussion

The phenotypic instability of bone marrow‐derived SCLCs is at issue with the intended use of these cells for nerve repair and regeneration after therapeutic transplantation. In this work, we demonstrate that commitment to the Schwann cell fate can be acquired by SCLCs in coculture with sensory neurons via contact‐dependent cues, mimicking the niche within developing DRGs. Although access to incipient neurons of developing DRGs harvested from human embryos is limiting, here we demonstrate the use of a human iPSC line to bypass the translational barrier. By various combinations of SMIs of cellular signaling pathways, we arrived at an 8‐day program that effectively and efficiently fostered differentiation of a human iPSC line into sensory neurons with respect to morphology, marker protein expression, electrophysiological properties, and amenability to myelination by Schwann cells.

To direct differentiation of iPSCs to sensory neurons, the following steps are critical: (a) exit from the pluripotent state, (b) differentiation into the lineages of embryonic tissues rather than extraembryonic tissues, (c) differentiation into ectoderm rather than primitive streak, (d) differentiation into neural cells rather than epidermal cells 29, and (e) differentiation into cells of the neural crest lineage rather than those of the central nervous system. Smad signals have multiple effects in each step of differentiation. Because TGFβ/Activin/nodal‐mediated Smad signaling maintains the pluripotency of human ESCs, inhibition of this signal results in induction of differentiation 30. BMP4‐mediated Smad signaling initiates differentiation into trophoblasts 31 or extraembryonic tissue 32, whereas nodal has an inhibitory effect on this step. Both BMP and Activin signals block ectodermal differentiation and promote the development of primitive streak 33. After ectodermal differentiation, BMP4 blocks neural differentiation and promotes epidermal development. Inhibition of BMP signaling promotes neural differentiation 34. Thus, concurrent inhibition of Smad signaling effected the differentiation of ESCs and iPSCs into specific cell types of the central nervous system 25, 35, 36, 37. Canonical Wnt signaling, which inhibits glycogen synthase kinase 3, performs roles in promoting neural crest formation in vertebrate development 38, 39, 40. Elevating Wnt signaling in the context of low Smad activity would divert early neuroectoderm away from an NPC fate toward a neural crest‐like identity 37. Activation of canonical Wnt signaling is also capable of instructing naive neural crest precursors toward the sensory neuron lineage. Notch signaling is also involved in maintaining cell pluripotency. Blocking Notch signaling can mediate the loss of pluripotency and drive neural differentiation of pluripotent stem cells. Based on the functions of SMIs, we chose to inhibit Smad with LDN193189, a potent inhibitor of BMP‐mediated Smad signaling 41, and A83‐01, a selective inhibitor of Activin‐mediated Smad signaling, which is more potent than the commonly used SB431542 42. RO4929097, a member of the secretase inhibitor family, was included to enhance neurogenesis by interfering with Notch signaling 36, 43. CHIR99021, acting as a Wnt agonist by selectively inhibiting glycogen synthase kinase 3β 26, was used in our protocol to accelerate neural crest formation and specification of sensory neurons 44, 45. SU5402, a potent inhibitor of FGFR1 signaling, was also applied not only to facilitate specification of neuroectodermal fate via PAX6 induction among human iPSCs but also to promote sensory neuron differentiation after neuroectoderm formation 46.

We then explored schedules of combinations of the SMIs for the derivation of sensory neurons from human iPSCs. In the 3‐step protocol, human iPSCs were induced to differentiate into PAX6‐positive NPCs via inhibition of both BMP and Activin A/nodal signaling with LDN193189 and A83‐01 for 5 days. Then, use of CHIR99021 in the context of A83‐01 effected the differentiation of the early PAX6‐positive population to p75^NTR^‐positive neural crest cell fates. Omission of LDN193189 had no major impact, indicating that active suppression of BMP signaling is not required at this stage (data not shown), as had been reported 37. Subsequently, treatment with RO4929097 and SU5402 in the context of CHIR99021 directed differentiation into neurons positive for markers of the sensory lineage. Because NCSCs and NPCs are developmentally derived from ectoderm progenitor cells, we reasoned that dual‐Smad inhibition in combination with Wnt activation would speed up the derivation of neural crest cells from iPSCs. Thus, treatment of iPSCs with CHIR99021, A83‐01, and LDN‐193189 resulted in a highly enriched p75^NTR^‐positive neural crest‐like population within 5 days. Subsequent exposure to CHIR99021, RO4929097, and SU5402 facilitated the conversion to sensory neurons. The 2‐step protocol took 12 days, in contrast to 14 days required for the 3‐step protocol.

Given that the starting population of iPSCs are likely heterogeneous in molecular states that are responsive to the differentiation signals, we speculated that the cocktail of SMIs could generate synergism in the differentiation program to sensory neurons. We therefore attempted a one‐step treatment of the iPSCs with a cocktail of the five SMIs. Under 6–8 days of treatment, the vast majority of cells progressively adopted neuronal morphology and expressed not only cytoskeletal protein markers of neurons but also the transcription factors, BRN3A, Islet, and NeuN, identifiable with sensory neurons. The resulting cells were, however, negative for NPC markers as well as neural crest cell markers, suggesting swift synchrony in the conversion from pluripotent stem cells to peripheral neurons. Follow‐up cultures of the derived sensory neurons in neural maintenance medium found expression of the three vesicular glutamate transporters 1, 2, and 3 (VGLUT1, VGLUT2, and VGLUT3) but not GABA (data not shown). A number of TUJ1‐positive cells expressed the synaptic vesicle proteins, synapsin and VAMP, suggesting the capacity of forming synapses in the neuronal network and releasing glutamate in excitatory neurotransmission. We have therefore sped up the time frame for generating the sensory neuron phenotype as an improvement over published protocols 12, 47. Whole‐cell patch‐clamp recordings indicated that the induced neurons could evoke single action potential‐like spikes in response to a depolarizing step current or multiple spikes with increasing intensity of the step current. That these spikes could be blocked with bath administration of TTX further suggests that the derived cells had differentiated into functional and electrically active neurons.

The level of NRG1 type III expressed on axons plays a key role in graded activation of ErbB2/3 heterodimers on the Schwann cell membrane and downstream signaling that culminate in fate choice between axonal ensheathment and myelination by Schwann cells 48, 49, 50, 51, 52. This was reinforced by our finding, in cocultures of the derived neurons and rat Schwann cells, that the nonmyelinating condition primed neurons with upregulated expression of NRG1 type III, whereas within 2 days of the transition to myelinating condition, contact‐mediated signaling between neuronal NRG1 type III and glial ErbB2/3 receptors was detectable as phosphorylated forms of ErbB2/3. Myelin‐like segments in alignment with neurites were evident in the myelinating cocultures. It remains unclear whether the neurite level of NRG1 type III determined Schwann cell ensheathment versus myelination or that, given time, the Schwann cells would have established mature myelin on the neurites. Admittedly, the status of compact myelin formation needs verification by analysis of electron micrographs.

Earlier efforts of in vitro derivation of SCLCs from rat BMSCs used a cytokine/growth factor cocktail 8, 9. However, the SCLCs lacked fate commitment and reverted to a fibroblast‐like phenotype upon withdrawal of differentiation‐inducing factors 53, and the phenotypically unstable SCLCs demonstrated limited capacity for remyelination. By coculture with embryonic rat DRG neurons, rat SCLCs were directed to fate commitment; consequently, the derived Schwann cells demonstrated robust capacity for remyelination in vitro 8 and in vivo functional recovery after transplantation as a Schwann cell‐seeded guidance channel bridging a critical gap in a rat model of sciatic nerve injury 10. More importantly, the iPSC‐derived sensory neurons generated in the present study can be used to replace rat DRG neurons in providing neural membrane effectors that switch the SCLCs to committed Schwann cells.

In sum, knowledge of key signaling pathways is instrumental in establishing culture conditions for the differentiation of human iPSCs along a neuroectoderm pathway for the generation of neuronal subtypes. Here we report that concurrent activation of canonical Wnt signaling under conditions of low global Smad signaling, as well as low Notch and FGFR1 signaling, is adequate for inducing rapid differentiation of human iPSCs into sensory neurons. This protocol is a major improvement over published methods in that the single‐step induction could conveniently be performed in chemically defined medium using small‐molecule compounds that are stable and cost‐effective. Populations of derived sensory neurons may then be used in the translation to a protocol whereby human bone marrow‐derived Schwann cells achieve fate commitment and meet safety requirements for autologous transplantation and remyelination therapy.

## Conclusion

We developed a highly efficient chemical protocol to differentiate human iPSCs into functional sensory neurons. We found that the derived sensory neurons expressed membrane‐bound cues that direct bone marrow‐derived SCLCs to fate‐committed Schwann cells with translational prospects in autologous transplantation and remyelination therapy. The iPSC‐derived sensory neurons will also be of utility in studies of sensory neuron biology, disease modeling, and drug screening.

## Author Contributions

S.C.: conception and design, experimentation, data analysis and interpretation, manuscript writing, final approval of manuscript; L.H.: experimentation and data analysis; Q.A.: conception; Y.‐S.C. and D.K.‐Y.S.: conception and design, data analysis and interpretation, manuscript writing, financial support, final approval of manuscript.

## Disclosures of Potential Conflicts of Interest

The authors indicated no potential conflicts of interest.

## Supporting information

Supporting Information
