# Supplementary material for: Human Induced Pluripotent Cell‐Derived Sensory Neurons for Fate Commitment of Bone Marrow‐Derived Schwann Cells: Implications for Remyelination Therapy
Source: Stem Cells Transl Med. 2016 Sep 14;6(2):369–81. doi: 10.5966/sctm.2015-0424 (PMC5442799; doi:10.5966/sctm.2015-0424)
Supplement: Supplementary file 1 — Supporting Information [file SCT3-6-369-s001.pdf]

Supplementary Figures and Table

Human iPS Cell-Derived Sensory Neurons for Fate Commitment of Bone Marrow-Derived Schwann Cells – Implication for Re-myelination Therapy - SA CAI et al.

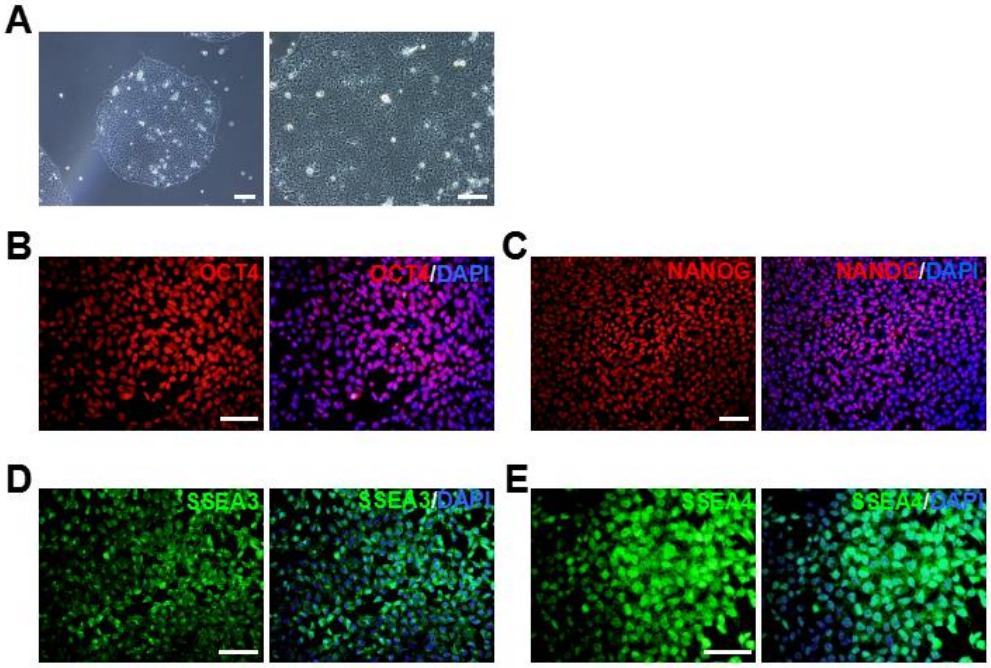

Figure S1. Characterization of induced pluripotent stem cells (iPSCs)

Phase-contrast images of human iPSC colonies grown on human embryonic stem cell (ESC)-qualified matrix (a). Fluorescent images of human iPSCs showing immunopositivities for OCT4 (b), NANOG (c), SSEA3 (d) and SSEA4 (e). Cell nuclei were visualized with DAPI. Scale bar=50µm for all panels.

Abbreviations: DAPI, 4, 6-diamidino-2-phenylindole.

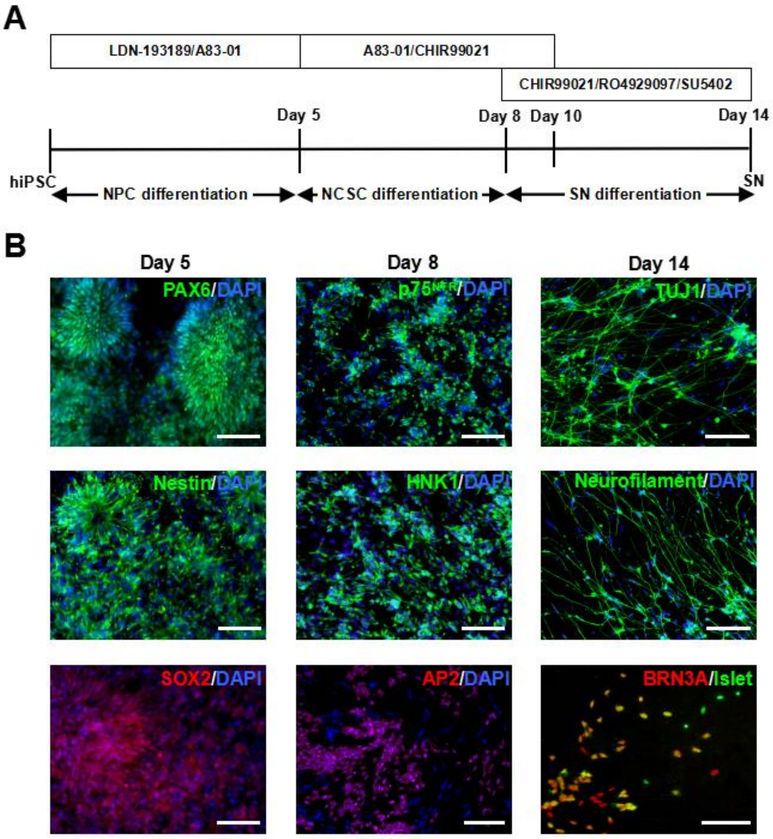

Figure S2. Differentiation of induced pluripotent stem cells (iPSCs) to sensory neurons via three-step protocol

A. Differentiation scheme of iPSCs to sensory neurons by three-step protocol.

B. In the condition of dual-Smad inhibition using LDN-193189 and A83-01 for 5 days, the expression of PAX6, nestin and SOX2 was robust in cultures, indicating the occurrence of neural progenitor cell (NPC) induction. Starting on day 5, Wnt activator CHIR99021 was added. Three days later, neural crest differentiation was observed based on p75^{NTR}, HNK1 and AP2 expression. In the context of CHIR99021, when Notch inhibitor RO4929097 and FGFR1 inhibitor SU5402 were added for 6 days, a number of cells expressed TUJ1, neurofilament, BRN3A and Islet, indicating sensory neurogenesis. The cell nuclei were visualized by DAPI. Scale bar=50μm for all panels.

Abbreviations: DAPI, 4, 6-diamidino-2-phenylindole.

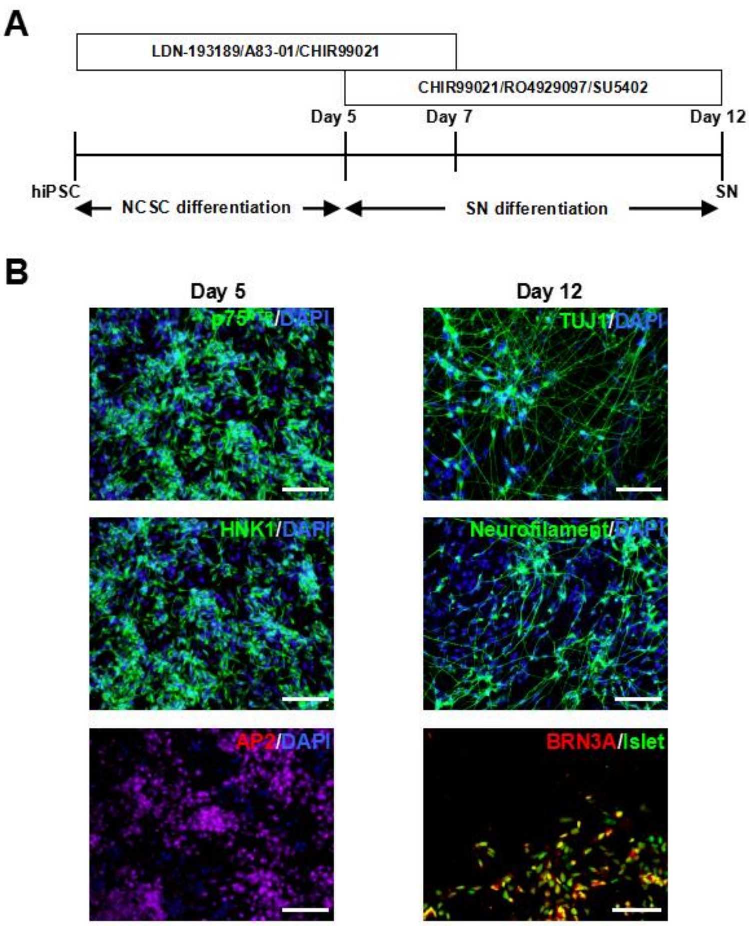

Figure S3. Differentiation of induced pluripotent stem cells (iPSCs) to sensory neurons via two-step protocol

A. Differentiation outline of iPSCs to sensory neurons by two-step protocol.

B. When exposed to the combination of LDN-193189, A83-01 and CHIR99021 for 5 days, neural crest could be identified based on $p75^{NTR}$, HNK1 and AP2 expression. When three inhibitors (CHIR99021, RO4929097, SU5402) were applied for 7 days, cells expressing TUJ1, neurofilament, BRN3A and Islet were widely present in cultures, indicating the specification of iPSC-derived neural crest stem cells (NCSCs) toward sensory neurons. The cell nuclei were visualized by DAPI. Scale bar=50μm for all panels.

Abbreviations: DAPI, 4, 6-diamidino-2-phenylindole.

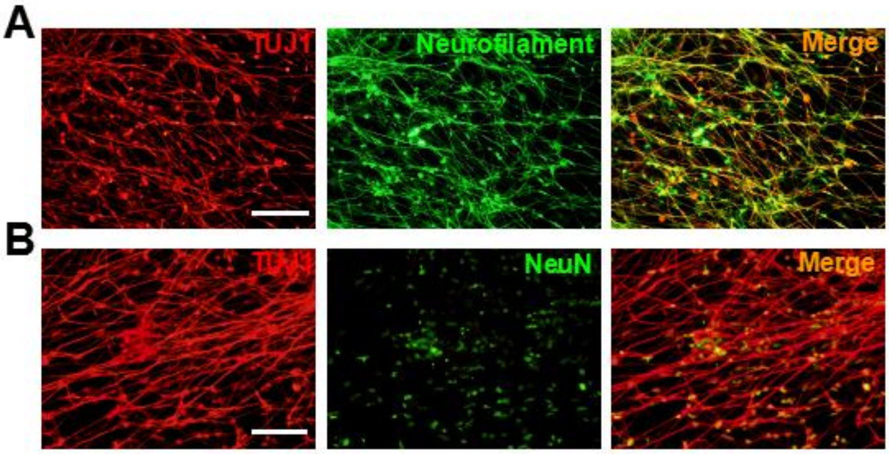

Figure S4. Differentiation of induced pluripotent stem cells (iPSCs) to sensory neurons via one-step protocol

Double-labeled immunocytochemistry showed TUJ1 and neurofilament (A), TUJ1 and NeuN (B) can be found expressed in the differentiated cells. Scale bar=50µm for all panels.

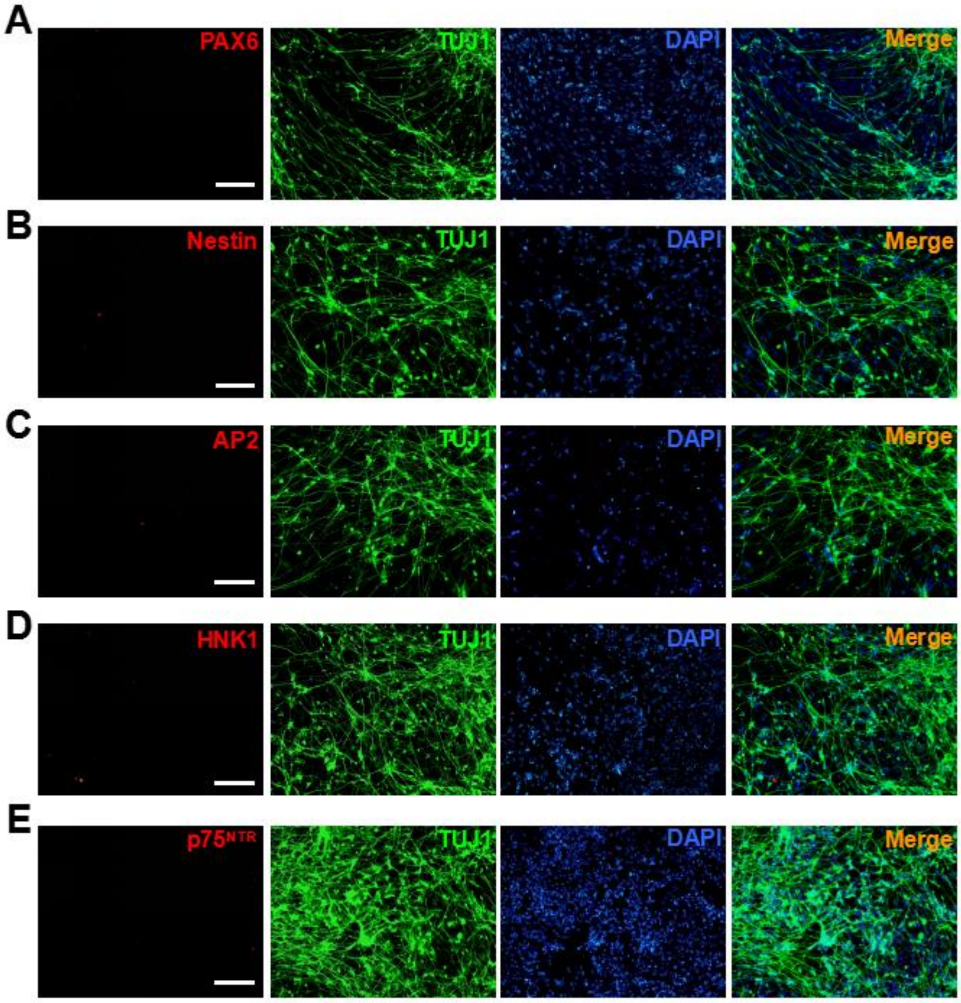

Figure S5. An occurrence of a swift conversion from induced pluripotent stem cells (iPSCs) to sensory neurons

Immunocytochemistry of neural progenitor cell (NPC) and neural crest stem cell (NCSC) markers. The resulted cells showed immunonegativity for PAX6 (a) and nestin (b); and also for AP2 (c), HNK1 (d) and $p75^{NTR}$ (e). Nuclei were visualized with DAPI. Scale bar=50μm for all panels.

Abbreviations: DAPI, 4, 6-diamidino-2-phenylindole.

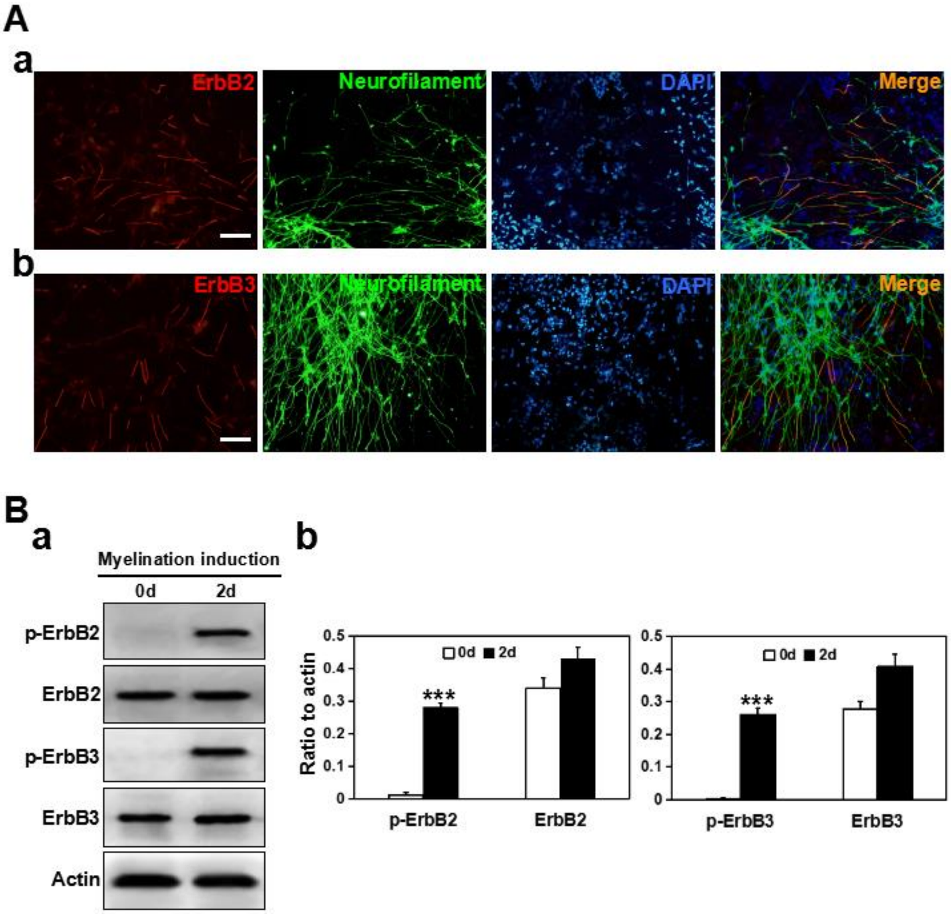
A
a
ErbB2
Neurofilament
DAPI
Merge
b
ErbB3
Neurofilament
DAPI
Merge
B
a
Myelination induction
0d
2d
p-ErbB2
ErbB2
p-ErbB3
ErbB3
Actin
b
0d 2d
Ratio to actin
0.5
0.4
0.3
0.2
0.1
0
***
p-ErbB2
ErbB2
p-ErbB3
ErbB3

Figure S6. ErbB2 and ErbB3 in rat Schwann cells when co-cultured with induced pluripotent stem cell (iPSC)-derived sensory neurons

A. Immunofluorescence for both ErbB2 (a) and ErbB3 (b) was observable in Schwann cells contacting the neurofilament-positive neuritic networks of derived sensory neurons in co-culture under myelinating condition for 14 days. Nuclei were visualized with DAPI. Scale bar = 50µm for all panels.

B. Expression levels of ErbB2/ErbB3 in co-cultures as revealed by Western blotting (a). Activation states of ErbB2 and ErbB3 were detected with use of activation state-specific antibodies (p-ErbB2, p-ErbB3). In the transition from day 7 under non-myelinating condition (0d) to a further 2 days under myelinating condition (2d), significant increases in p-ErbB2 and p-ErbB3 were observed against the levels before myeliantion induction (b). ***, $p<0.005$, p-ErbB2 and p-ErbB3 on day 2 after myelination induction vs. p-ErbB2 and p-ErbB3 on day 0 before myelination induction.

Abbreviations: DAPI, 4, 6-diamidino-2-phenylindole; p-ErbB2, phospho-ErbB2; p-ErbB3, phospho-ErbB3.

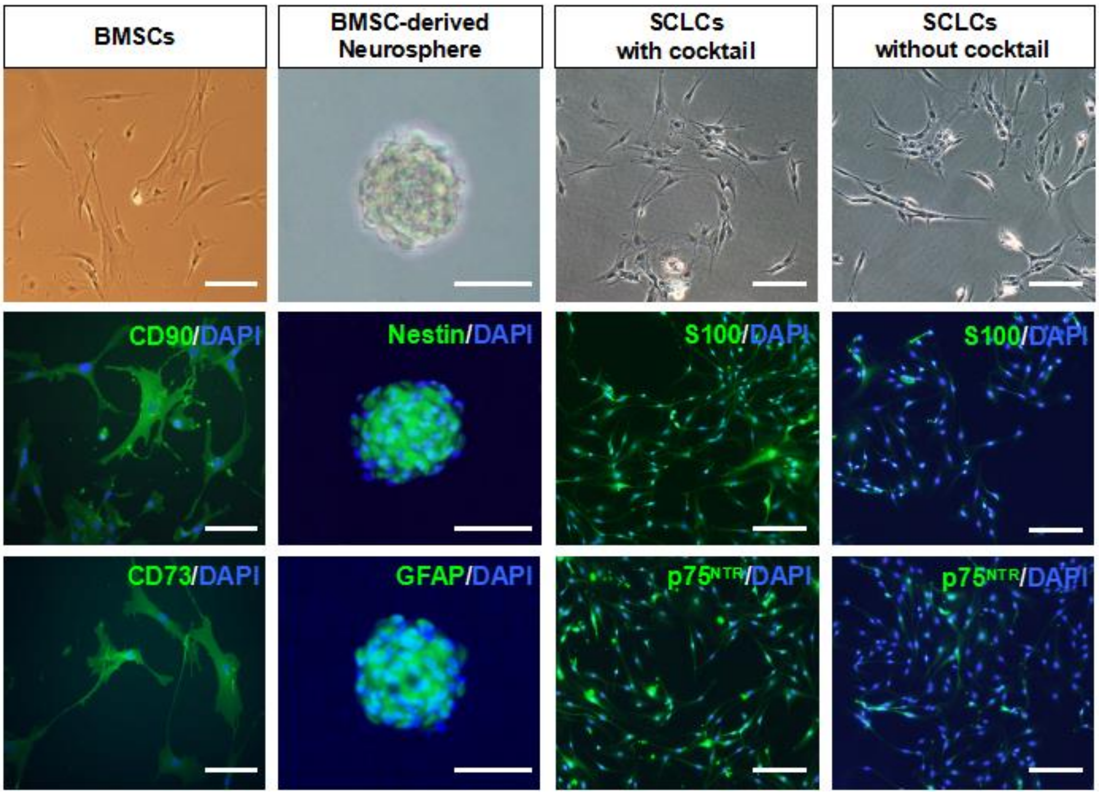

Figure S7. Generation of Schwann cell-like cells (SCLCs) from adult rat bone marrow stromal cells (BMSCs)

Neuroectodermal progenitors among CD90- and CD73-positive BMSCs were selectively expanded and formed nestin- and GFAP-positive neurospheres. S100- and p75^{NTR}-positive SCLCs were derived from neurospheres in an induction cocktail of gliogenic factors. The SCLCs were not able to maintain the expression of Schwann cell markers after the induction cocktail had been removed from the cultures. Scale bar=100µm for all panels.

Table S1. Polymerase Chain Reaction Primer Pairs

Gene	Sequence	Product size (bp)	Anneal (^{o}C)	Cycle no.
TUJ1	5'-AGATGTTCGATGCCAAGAA-3'	164	58	35
	5'- GGATCCACTCCACGAAGTA -3'			
Islet	5'-GTAGAGATGACGGGCCTCAG-3'	234	59	35
	5'-TTTCCAAGGTGGCTGGTAAC -3'			
Peripherin	5'- AAGACGACTGTGCCTGAGGT-3'	116	59	35
	5'- TGCTCCTTCTGGGACTCTGT -3'			
BRN3A	5'-ACTCAGCCAGAGCACCATCT -3'	277	60	35
	5'- TTTGAGGTCCAGTTTCTCGG -3'			
GAPDH	5'-TTAGCACCCCTGGCCAAGG-3'	531	58	30
	5'-CTTACTCCTTGGAGGCCATG-3'			
